# A Case of Intranasal Hemangioma and Concurrent Tetracycline-induced Ulcerative Gastritis in Dogs

**DOI:** 10.4103/0971-6580.68348

**Published:** 2010

**Authors:** H. S. Banga, S. Deshmukh, R. S. Brar, P. D. Gadhave, S. G. Chavhan, H. S. Sandhu

**Affiliations:** Department of Veterinary Pathology, College of Veterinary Science, GADVASU, Ludhiana - 141 004, Punjab, India; 1 Department of Pharmacology and Toxicology, College of Veterinary Science, GADVASU, Ludhiana - 141 004, Punjab, India

**Keywords:** Dogs, doxycycline, gastritis, histopathology, tetracyclines

## Abstract

Incidence of drug-induced gastritis and ulceration in human medicine is well established. Besides, unilateral hemangioma, a unique concurrent case of tetracycline induced gastric toxicity in a dog, characterized by gastritis and ulceration is being reported here. Grossly, the appearance of gastric ulcers mimicked the appearance of Italian pizza. Histological examination further supported drug-induced etiology in this case. This is probably the one of the few cases in the annals of veterinary medicine to be documented as drug-induced gastric toxicity in dog.

## INTRODUCTION

Incidence of intranasal tumor(s) in dogs accounts for very rare number of cases;[1] out of the cases reported, benign kind of intranasal vascular tumors are extremely rare. However, malignant types of intranasal vascular tumors credit little higher incidence rate as compared to the benign one. The incidence of such intranasal tumors is common in dogs residing in urban locality where a contact to environmental carcinogens and pollutants is more.[2] As a feature, canine hemangioma has been mainly reported in skin, oral mucosa, tongue, spinal cord, kidney, liver, and spleen.[3,4] However, hemangioma as an intranasal tumor appears as a rare benign lump comprising blood vessels in inner aspects of nasal cavity, with continuous or intermittent oozing of blood, often creating confusion to sizeable number of disease conditions in canine clinical practice. Most commonly hemangioma and hemagiosarcoma are associated with blood clotting affects with decreased platelet count and increased blood clotting times, sometimes insinuating the condition for autoimmune-mediated hemolytic anemia, immune-mediated thrombocytopenia, and in case of nasal involvement, with canine ehrlichiosis. At times, such a false simulating condition of the disease results in incorrect diagnosis, resultantly faulty regimen of medication, leading to various secondary effects to the body, and sometimes death, of an animal.

Gastric ulceration following disruption to gastromucosal barrier in dogs due to excessive antibiotic therapy is an uncommon incidence. Most commonly, ulceration in gastric mucosa of dogs appears due to excessive usage of nonsteroid anti-inflammatory drugs (NSAIDs) and corticosteroid as secondary to primary therapy. At many times, the gastric ulceration would also appear due to regurgitation of bile acids, which invokes gastrin release, leads to hypersecretion of gastric acids, and causes ulceration. Medication with tetracycline(s) in the form terramycin and doxycycline therapy is the most preferred choice for the clearing of the chronic infection of *Ehrlichia canis* from dogs[5,6] resulting in gastric ulcers in dogs almost uncommon. Doxycycline as an incriminating etiology for the induction of gastric ulcers in human being is reported in few cases.[7]

## MATERIALS AND METHODS

Here, we encountered one such typical isolated case of unilateral intranasal hemangioma with history of nasal bleeding from nostrils and subcutaneous hemorrhages at the level of abdomen, along with a record of high fever from a intact male Labrador dog aged 7 years and 6 months. The dog was suspected to be having canine ehrlichiosis and accordingly treated with doxycycline, which resulted in drug-induced gastric ulceration. In fact, the thorough postmortem examination of the dog revealed the presence of unilateral nasal hemangioma and gastric ulceration. This present study hopes to open an insight into the drug-induced toxicity and demands insurance in terms of diligence and meticulous examination of animal to comprehend the possible etiology of the disease to avoid such misincidence in the near future.

The initial hematobiochemical test carried out in this case revealed macrocytic hypochromic anemia, marked leucopoenia, and thrombocytopenia with borderline increase in the value of alkaline phosphatase enzymes. The blood sample collected for hemoprotozoan infection did not reveal any conclusion for ehrlichiosis. In this case, the lack of demonstration of organism in smear could be mistakenly considered for ehrlichiosis as an infection with low intensity. This kind of observation with the absence of organism in the blood smear due to low infection rate was earlier reported in literature many a times. Following consideration of such report, the animal was put on prolonged antibiotic therapy with terramycin (oxytetracycline) along with adrenaline and stergyl. The drug terramycin was continued for 7 days i.v. single shot. The bleeding from nostril stopped immediately the next day, but no respite from pyrexia could be observed, till death. Next, the regimen for antibiotic therapy was shifted to doxycycline with a dose of 5 mg/kg PO b.i.d, instead of s.i.d, which again lasted for closely 8 days. The hematological study carried out at subsequent phases after antibiotic treatment (doxycycline) revealed no further improvement in the values of parameters.

Gradually, the condition of the animal deteriorated and was considered for blood transfusion. Even after this effort, the animal collapsed, died, and was sent to necropsy annexe of Department of Veterinary Pathology, COVS, GADVASU, for detailed necropsy.

## RESULTS AND DISCUSSION

**Figure 1:**
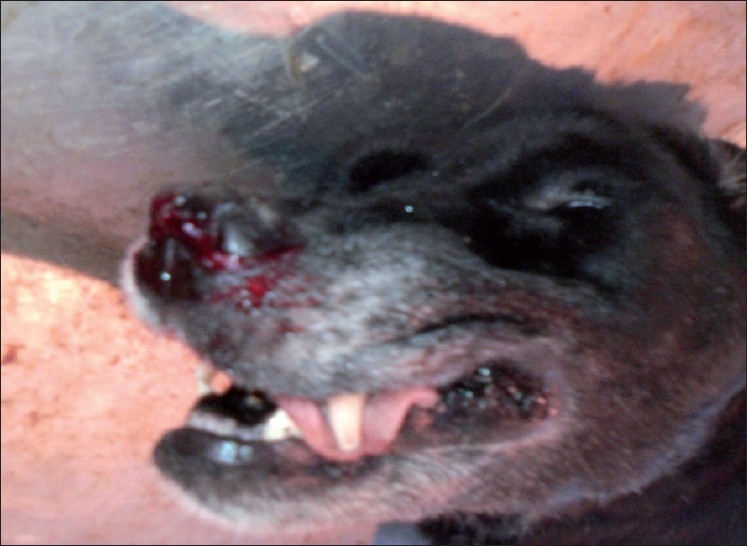
Nasal epistaxis

**Figure 2:**
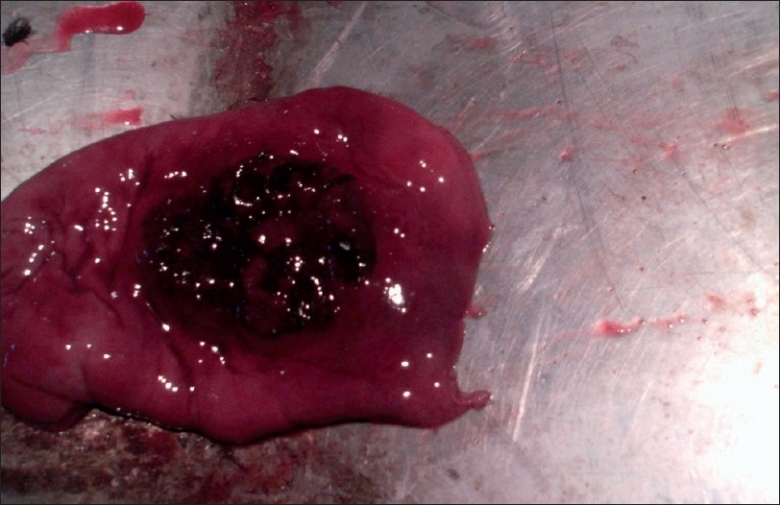
Gastric ulceration, a large reddish plaque-like growth with slightly raised outlines was appreciated besides congestion and thickening of wall of stomach

At the very initial observation, the carcass revealed epistaxis [Figure 1], i.e., blood-stained nasal mucosa with an involvement of nasal orifices and around mouth. Following necropsy, the gross examination of the nasal cavity exhibited a small nonfulminating bluish-purple colored growth nearing to nasal turbinate having variegated black granular contents. On opening the respiratory system, particularly the lungs revealed marked edema and the trachea showed blood stained plaques attached to mucosal lining. Liver appeared slightly pale in color, giving a semblance to initial phase of jaundice. On incision to the stomach, a large reddish plaque like growth with slightly raised outlines was appreciated [Figure 2], and mimicked an Italian pizza with chopped vegetables over, indicated gastric ulceration. The cut surface of plaque revealed sprouted black colored granules-like growth. The gastric ulceration in the present study is not only due to overdosing of doxycycline, but can be because of prolonged usage of terramycin, which contains oxytetracycline as a major ingredient. The kidney was apparently normal in the gross observation. However, the adrenal over kidney appeared slightly enlarged. At the last, the apparently significant tissue samples were taken into 10% formalin for initial fixation and subsequent histopathological examination. The fixed tissues were further subjected to paraffin-embedding technique and cut tissue sections were stained with hematoxylin and eosin stain for light microscopic studies.[8]

**Figure 3:**
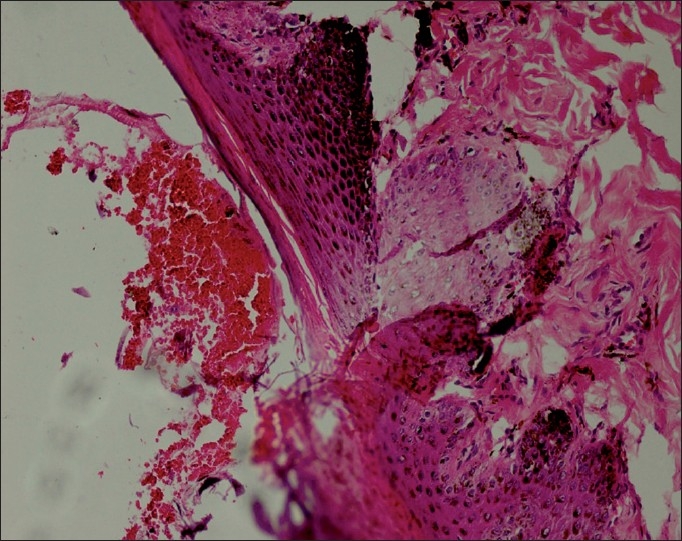
Cavernous hemangioma lesions attached to the nasal epithelium (H and E ×150)

**Figure 4:**
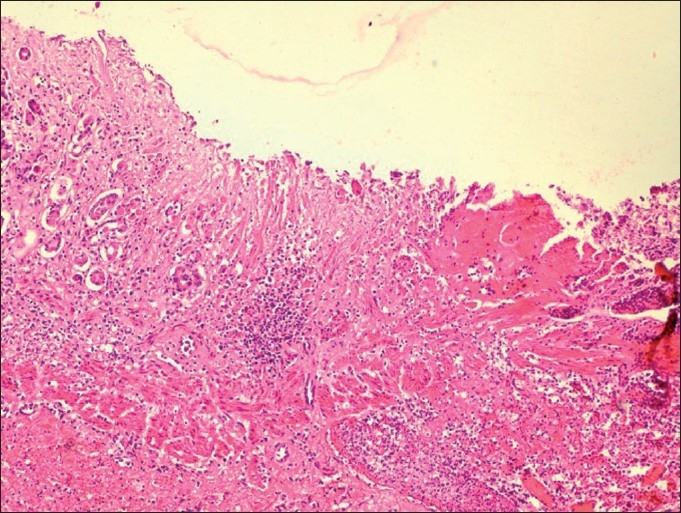
Chronic ulcerative gastritis revealed complete replacement of mucosal lining by large areas of necrotic debris and marked destruction of gastric glands, fibroplasia (H and E ×150)

**Figure 5:**
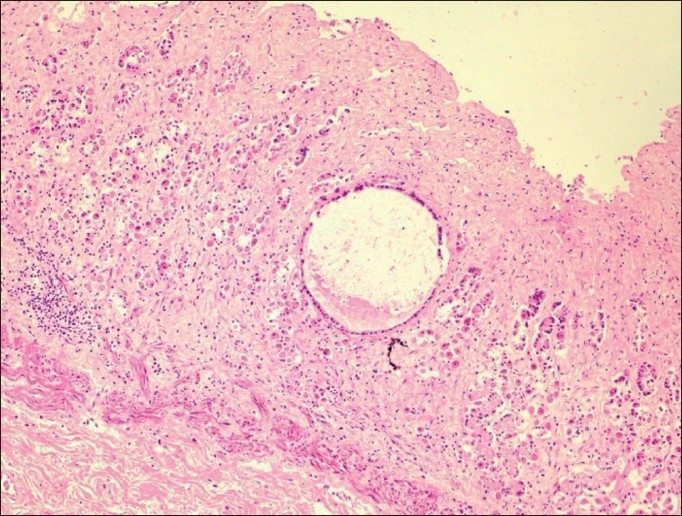
Chronic ulcerative gastritis with evidence of mucocele and with population of lymphomononuclear cells (H and E ×150)

On microscopic examination, the nasal cavity testified the presence of cavernous hemangioma lesions attached to the nasal epithelium [Figure 3]. The lesion was characterized by multiple anatomizing blood-filled space network. The much extent of hemangioma appeared completely occluding the nasal cavity. The lungs contained extensive congestion, hemorrhages, and wide zone of pulmonary edema. Overdosing of doxycycline responsible for pulmonary edema has been already reported by Yeruham *et al*.[9] in calves. Following microscopic intervention to the gastric tissues, the confirmatory evidence of chronic ulcerative gastritis was made [Figure 4]. The luminal end revealed complete replacement of mucosal lining by large areas of necrotic debris and marked destruction of gastric glands. The ulcerative areas appeared well marginated by occasional numbers of atrophic gastric glands, which were interspersed amidst the zone of fibroplasia along with evidence of mucocele [Figure 5] and abundant population of lymphomononuclear cells. This classical microscopic finding greatly supports toward drug-induced etiology of the gastric ulcers owing to doxycycline overdosing and prolonged terramycin therapy. Oxytetracycline in the form of terramycin is also found to be corrosive to gastrointestinal mucosal lining. Recently Akbayir *et al*.[7] demonstrated doxycycline as potent ulcerogenic drug in causing gastric ulcers in a female patient. The ulcerogenic potential of tetracycline group of drugs described earlier by Carlborg and Densert[10] in the form of circumferential ulcer in esophageal mucosa of cat confirms its corrosive effects on mucosal lining and is very similar to the gross and microscopic observation, made in the present case. The submucosal area of stomach exhibited peculiar findings characterized by noticeable zone of congestion and large area of hemorrhages along with severe edema.

**Figure 6:**
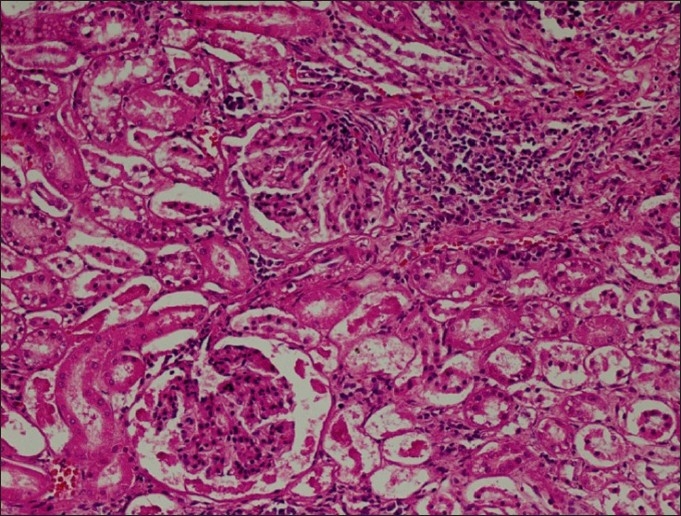
The kidney revealed marked interstitial nephritis featured with many areas of mononuclear cell infiltration

The heart specifically characterized by marked myocardial hemorrhages with mild to moderate disruption in the structural array of myocardial fiber, which could be due to overdosing of doxycycline. Almost closely to this observation, Yeruham *et al*.[9] indicated myocardial necrosis suggesting cardiotoxic effects of doxycycline overdosing. In spleen, multifocal areas of golden yellowish pigmented zone of hemosiderosis due to hemorrhages were seen. At places, large areas of infarcts (red infarct) surrounded by a zone of inflammation was also noticed in spleen. The kidney revealed marked interstitial nephritis [Figure 6] featured with many areas of mononuclear cell infiltration (MN cell), mainly at corticomedullary junction. Moreover, the evidence of tubular degeneration characterized by marked vacuolation of tubular epithelium at the cortex was also noticed. The details of nephrotoxic event made in this study could be well corroborated with the feature of oxytetracycline-mediated nephrotoxicity reported by various workers[12] and indicates the possible pathological effect in the nephrons due to terramycin.
